# Additively Manufactured NdFeB Polyphenylene Sulfide Halbach Magnets to Generate Variable Magnetic Fields for Neutron Reflectometry

**DOI:** 10.1089/3dp.2020.0340

**Published:** 2022-08-03

**Authors:** Tej Nath Lamichhane, Timothy R. Charlton, Brian Andrews, Devanshi Malaviya, Arjun K. Pathak, Haile Ambaye, Mathieu Doucet, Valeria Lauter, John Katsaras, Brian K. Post, Mariappan Parans Paranthaman

**Affiliations:** ^1^ Chemical Sciences Division, Oak Ridge National Laboratory, Oak Ridge, Tennessee, USA.; ^2^ Neutron Scattering Division, Oak Ridge National Laboratory, Oak Ridge, Tennessee, USA.; ^3^ Department of Physics, Buffalo State, The State University of New York (SUNY), Buffalo, New York, USA.; ^4^ Shull Wollan Center, Oak Ridge National Laboratory, Oak Ridge, Tennessee, USA.; ^5^ Department of Physics and Astronomy, University of Tennessee, Knoxville, Tennessee, USA.; ^6^ Manufacturing Science Division, Oak Ridge National Laboratory, Oak Ridge, Tennessee, USA.

**Keywords:** printed NdFeB-bonded magnets, Halbach arrays, PPS polymer NdFeB composite magnets, neutron reflectivity

## Abstract

Halbach arrays are the most efficient closed structures for generating directed magnetic fields and gradients, and are widely used in various electric machines. We utilized fused deposition modeling-based Big Area Additive Manufacturing technology to print customized, compensated concentric Halbach array rings, using polyphenylene sulfide-bonded NdFeB permanent magnets for polarized neutron reflectometry. The Halbach rings could generate a 0 ≤ *B ≤* 0.30 T field, while preserving 90% polarization of an axial neutron beam. Polarized neutron beams are used to study a wide range of structural and magnetic phenomena spanning physics, chemistry, and biology. In this study, we demonstrate the effectiveness of additive manufacturing for producing prototype Halbach arrays, characterize their magnetic properties, and generated magnetic fields, and discuss the conservation of neutron beam polarization as a function of magnetic field.


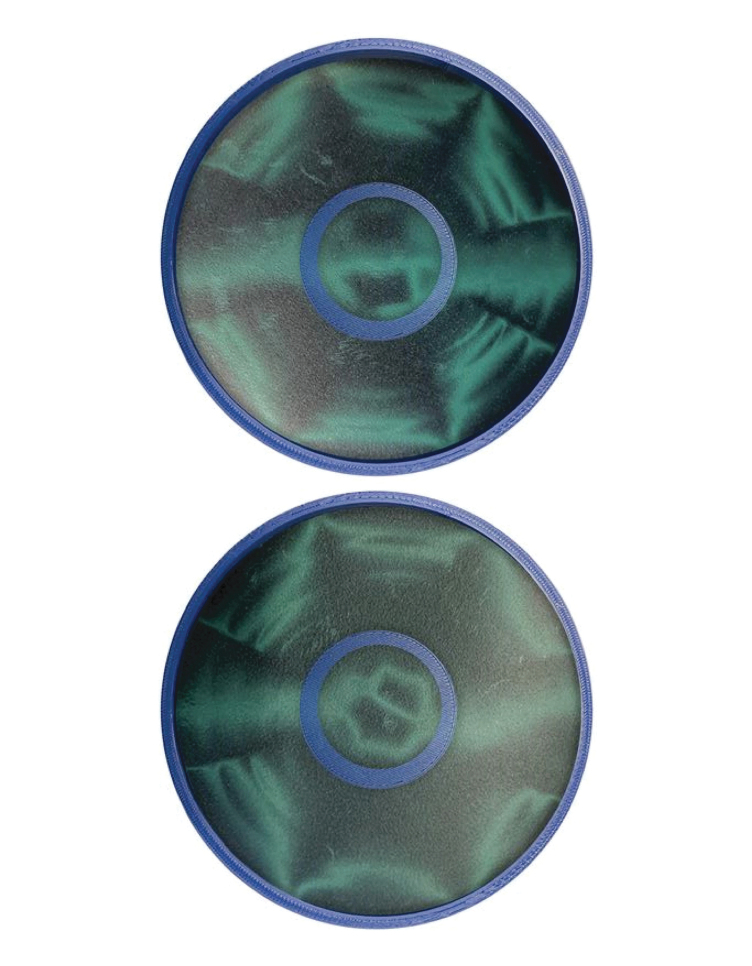


## Introduction

The proof of concept for generating a unidirectional magnetic field using vector addition of magnets was first demonstrated by Mallinson.^1^ Later, Halbach utilized this principle to generate multipole magnets,^2^ which are vital components in linear particle accelerators,^3,4^ free electron lasers,^5^ nuclear magnetic resonance,^6^ portable magnetic resonance imaging,^7^ electrical synchronous motors,^8^ Halbach-array permanent magnet (PM) motors,^9^ direct drive generators,^10^ actuators,^11–13^ high-gradient magnetic filtration in mineral oil purification,^14^ and many other applications. Halbach arrays can be used to focus or defocus spin-bearing particles and also splitting or merging their beams.

**FIG. 1.**
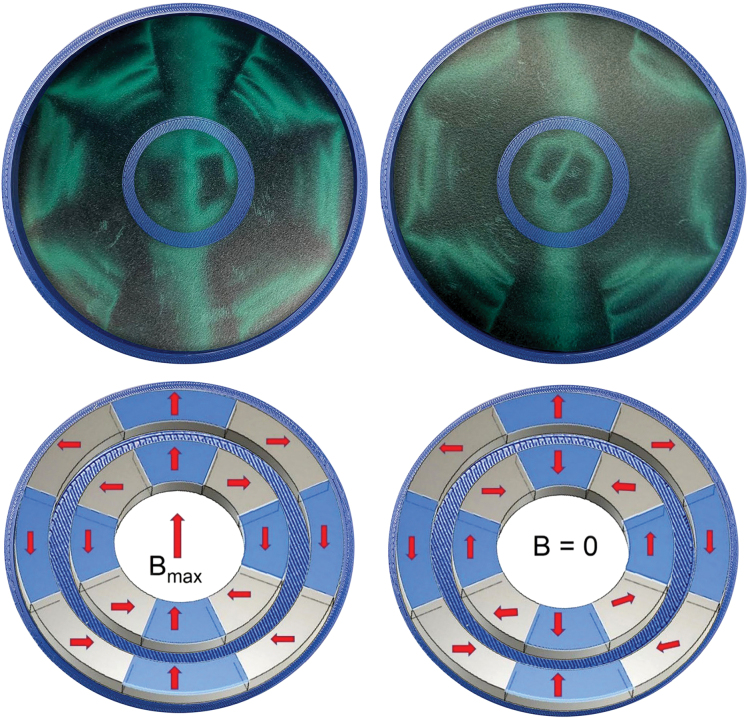
Schematic illustration of the pattern of magnetization used to generate a large dipolar field and zero field in nested Halbach arrays (*bottom*); image of the nested Halbach array with the magnetic film viewing on top showing magnetic field strength. Color images are available online.

A recent publication—and references therein—discusses the use of Halbach arrays in a CBETA (Cornell-Brookhaven National Laboratory [BNL] Energy Recovery Linac Accelerator) project.^4^ Moreover, due to their self-supported and closed geometry, Halbach rings are also used in frictionless ball-bearings and high-speed, low-inertia electric motors. The magnetic field produced in nested Halbach arrays is the vector sum of the magnetic fields from the individual rings. Magnetic fields can be increased to a maximum field strength if both rings are in a parallel arrangement (Fig. 1a) and minimized when the rings are in antiparallel positions (Fig. 1b)—the magnetic field can be varied by rotating both rings. Currently, single and concentric Halbach structures are actively being explored for emission-free magnetocaloric cooling.^15–17^

Recently, there has been an increased demand for high-flux PMs, namely Dy-doped Nd_2_Fe_14_B (Nd-Fe-B) magnets, where Dy and Nd are currently in limited supplies. This increased demand stems from a significant improvement in the properties of rare-earth-based, high-flux PMs, a development that has revolutionized the manufacturing of efficient and portable electrical devices.^18^ Unfortunately, current industrial-scale production methods, such as injection molding, compression molding, and powder sintering, can only provide a limited number of PM shapes and sizes. Also, due to the manufacturing processes used, much of the critical magnetic material is wasted. However, customization of complicated PM-based electric machines is currently beyond the scope of commercialization when using traditional manufacturing techniques.

The Halbach array is the most efficient PM assembly^19^ and presents an attractive solution to the current magnet supply problem, where the internal magnetic field scales with the product of average magnetization and the logarithm of the ratio of the external and internal radii of the ring. However, there are several challenges associated with manufacturing Halbach arrays, such as mechanical strength, field inhomogeneity, material criticality, eddy current losses, and radiation damage.^20–22^ To make the Halbach array more commonplace, it is, therefore, necessary to minimize such challenges. For example, the issue of field inhomogeneity can be addressed with more stringent criteria for the dimensions and homogeneity of the fabricated PMs and their arrangement in Halbach arrays. In addition, shimming can be used to improve magnetic field homogeneity.^23^

A key benefit of additive manufacturing (AM) processes is their ability to print magnets in a near net-shape configuration using computer-aided design (CAD) models without any tooling.^21,22^ The AM method uses a layer-by-layer addition of material to manufacture complex and highly customized assemblies. It, therefore, lends itself ideally to situations where rapid prototyping is needed, thus accelerating fundamental research and development. Importantly, fused deposition modeling (FDM) is an AM approach that could be useful for creating the required geometry for a Halbach array, without the loss of valuable magnetic materials. FDM can print the required sections individually or print the complete geometry that can be sliced later. Importantly, it is technologically advantageous to print bonded PM to reduce eddy current losses^20,21^ and provide the necessary mechanical properties needed for motor and Halbach array applications.

We additively manufactured a Halbach array capable of generating a variable magnetic field that, in conjunction with a polarized neutron beam, can be used to induce neutron contrast in magnetic multilayers, heterostructures, and biological samples. For example, neutron reflectometry is routinely used to study the one-dimensional structure of thin films with subnanometer resolution.^22^ By leveraging the magnetic moment of the neutron, polarized neutron reflectometry allows one to probe the magnetic structure of materials.^24,25^ By combining a magnetic layer of known structure into an otherwise unknown film structure, one can extract the phase information with polarized neutron reflectometry,^24–26^ thus enabling the direct solution to the one-dimensional, real space structure of the adsorbed thin film.^27–31^

A novel prototype Halbach ring magnet has been designed for the magnetism reflectometer^32–34^ at the Spallation Neutron Source (SNS) located at Oak Ridge National Laboratory (ORNL), using the Big Area Additive Manufacturing (BAAM) printer located at ORNL's Material Demonstration Facility. Polyphenylene sulfide (PPS) is reported to be more resistant to *γ*-rays^35^ and electron-beam exposure than most other polymers.^36^ Although there is no specific neutron damage study comparing nylon and PPS, we selected PPS-bonded Nd-Fe-B PMs to construct the proof-of-principle Halbach arrays for neutron reflectometry.

We successfully printed NdFeB-PPS magnets and fabricated a prototype Halbach array using two nested Halbach rings and then demonstrated polarization conservation using a traverse polarized neutron beam. In the Introduction section, we describe the Halbach array and its application to unambiguously determine the phase information in a polarized neutron reflectometry experiment. In the Experimental Details section, the Halbach disc printing, characterization of the magnetic properties of the printed material, and the fabrication of the Halbach arrays are discussed. In the Results and Discussion section, we present and discuss the results in four different subsections. The Magnetic Properties subsection summarizes the magnetic properties of PPS-bonded NdFeB magnet.

In the Field Characterization and Design of Nested Halbach Arrays subsection, we describe the field characterization of the parent Halbach array and its partition into nested Halbach arrays. The Electromagnetic Simulations of Additively Manufactured and Magnetized Halbach Arrays subsection illustrates the flux lines and estimates the magnetic field using electromagnetic simulations for perfectly printed Halbach arrays. The observed discrepancies between experiment and modeling are explained in terms of potential imperfections. Finally, in the Demonstration of Conservation of Neutron Beam Polarizability with the Compensated Halbach Discs subsection, we demonstrate the conservation of neutron beam spin polarization using the BL4A neutron reflectometer located at SNS. In the Conclusions section, a short summary and conclusions are presented.

## Experimental Details

### AM of Halbach

**FIG. 2.**
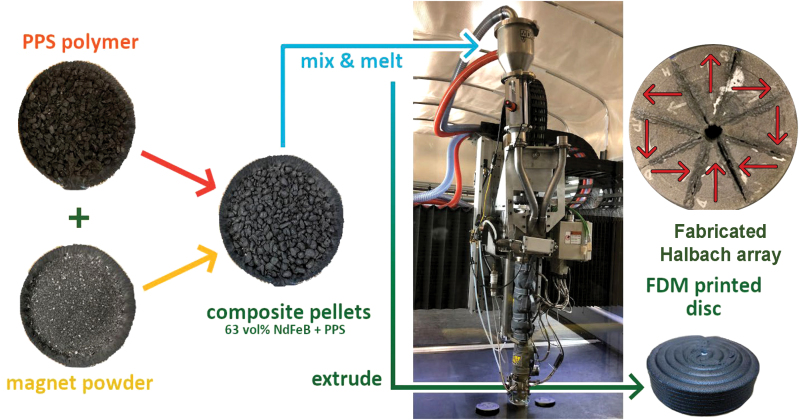
Schematics illustrating the FDM process for NdFeB PPS-bonded magnets. A Halbach array with an ID of 11 mm and OD of 110 mm (*top right*) was first characterized and then divided into two nested Halbach rings. The red arrows show the magnetization direction of the different sections of the printed magnets. FDM, fused deposition modeling; ID, inner diameter; OD, outer diameter; PPS, polyphenylene sulfide. Color images are available online.

NdFeB-PPS composite pellets with a 63:37 volume % ratio optimized for extrusion (Integrated Magnetics, Part number: L3082) were used to print the three-dimensional (3D) cylindrical discs needed for the Halbach arrays, as shown in Figure 2. The composite pellets were prepared by mixing PPS beads, NdFeB magnet powder, and a small amount of plasticizer. The pellets were then extruded through a five-zone polymer extruder barrel, with the different zones maintained at 305°C, 316°C, 321°C, 321°C, and 321°C, respectively, the fifth and final zone was at the tip. The heating elements are resistive band heaters that heat each zone individually by placing outside the extruder barrel. Thermocouples are used to measure the temperatures at each zone, a thermocouple was placed in the melt stream to measure the extrusion temperature. The pellets were fed using a single screw pellet dispenser into the BAAM printer (Cincinnati Inc., Harrison, OH). To print the composite cylinder, a cylindrical CAD model with a 110 mm diameter and 25 mm thickness was designed using Fusion 360. The conditions for NdFeB-PPS polymer printing are reported in Paranthaman et al.^21^ A 5.08-mm-diameter (0.2 inch) printer nozzle was selected and heated up to 325°C to dispense the composite layer, which was pressed with a special hoop-like design at the end of the printer nozzle (Fig. 2). The carbon fiber printing bed was kept at 95°C to maintain a temperature gradient between successive layers for print stability.

The printed disc was sliced into eight segments and magnetized at room temperature. The magnetized segments were then assembled to form the Halbach disc, as shown in Figure 2 (top right). The assembled Halbach array was drilled to have an 11 mm internal bore, matching the neutron beam size used for experiments. After complete characterization of the large disc's magnetic field, two inner and outer Halbach rings were cut from the large disc (discussed in the section below). The magnetic properties of the printed parts were measured with the physical property measurement system (PPMS) (Quantum Design, USA), and the magnetic field and its components were measured using a Hall probe (Metrolab Three-axis Hall-magnetometer).

## Results and Discussion

### Magnetic properties

**FIG. 3.**
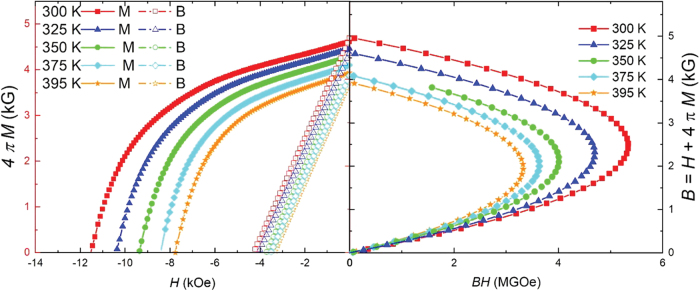
Variation of the demagnetization quadrant as a function temperature. The reversible temperature coefficients for remanence and coercivity were determined to be −0.16/°C and −0.34/°C, respectively, for the printed magnets. Color images are available online.

The room temperature magnetic hysteresis behavior, *M(H)*, of the starting composite pellets and the printed magnets was almost identical (not shown here), showing that the FDM printing did not degrade the NdFeB-PPS composite magnets. *M(H)* of the second quadrant at 300°C, 325°C, 350°C, 375°C, and 400°C is shown in Figure 3. The temperature coefficients for remanence, *α*, and coercivity, *β*, of the printed magnets were then determined (Table 1) using the following formulae:

**Table 1.** Comparison of the Temperature Coefficients of Different Permanent Magnets

Magnets	α (/°C)	β (/°C)	Temperature range (°C)
Sintered NdFeB	−0.12	−0.50 to −0.65	20 to 100+
Sm_2_Co_17_	−0.35	−0.20	20 to 120
SmCO_5_	−0.04	−0.40	20 to 120
AlNiCo	−0.02	−0.01	20 to 120
Bonded NdFeB	−0.07 to −0.13	−0.40	20 to 120
FDM anisotropic nylon NdFeB	−0.09	−0.53	20 to 120
FDM PPS NdFeB (this work)	−0.16	−0.34	20 to 120

From Constantinides^37^ and Gandha et al.^38^

FDM, fused deposition modeling; PPS, polyphenylene sulfide.

(1) $$\alpha =\frac{B_{rem}\left(400K\right)-B_{rem}\left(300K\right)}{B_{rem}\left(300K\right)}\times \left(400-300\right)\times 100\%$$

(2) $$\beta =\frac{H_{C}\left(400K\right)-H_{C}\left(300K\right)}{H_{C}\left(300K\right)}\times \left(400-300\right)\times 100\%,$$

where *B_rem_* and *H_C_* are the remanence and coercivity, respectively, at given temperatures. Table 1 compares the temperature coefficients of conventional and FDM-based additively manufactured PMs. The *B_rem_* and *H_C_* values at 300°C were used in the analysis of the magnetic fields of the AM-fabricated rings.

### Field characterization and design of nested Halbach arrays

The magnetic field at the center of Halbach disc was determined to be 0.60 T, using a Hall probe. The effective magnetic field at the center was then estimated using the following formula:

(3) $$B\left(r=0,z=0\right)=B_{rem}\left[ln\frac{r_{ex}}{r_{in}}+\frac{z_{0}}{2\times \sqrt{z_{0}^{2}+r_{in}^{2}}}-\frac{z_{0}}{2\times \sqrt{z_{0}^{2}+r_{ex}^{2}}}-ln\left(\frac{z_{0}+\sqrt{z_{0}^{2}+r_{ex}^{2}}}{z_{0}+\sqrt{z_{0}^{2}+r_{in}^{2}}}\right)\right]$$

where *B_rem_* is the remanent magnetization, *z*_0_ is the half thickness of the cylinder, and *r_in_* and *r_ex_* are the internal and external radii, respectively, of the Halbach cylinder.^16^

(4) $$0.60=B_{rem}\left[ln\frac{55}{5.5}+\frac{12.5}{2\times \sqrt{12.5^{2}+5.5^{2}}}-\frac{z_{0}}{2\times \sqrt{12.5^{2}+55^{2}}}-ln\left(\frac{12.5+\sqrt{12.5^{2}+55^{2}}}{12.5+\sqrt{12.5^{2}+5.5^{2}}}\right)\right]$$

Solving the equation, we obtained *B_rem_* = 0.357 T, which is very close to 90% of the room temperature magnetization remanence (0.40 T) for the printed material with magnetization 0.45 T—the 10% difference is attributed to material homogeneity and insufficient magnetization field used to align the different Halbach sections. The close agreement between the analytically predicted field parameters and the measured magnetic field of the additively manufactured Halbach opens possibilities for quality-controlled complex PMs for electric machines.

**FIG. 4.**
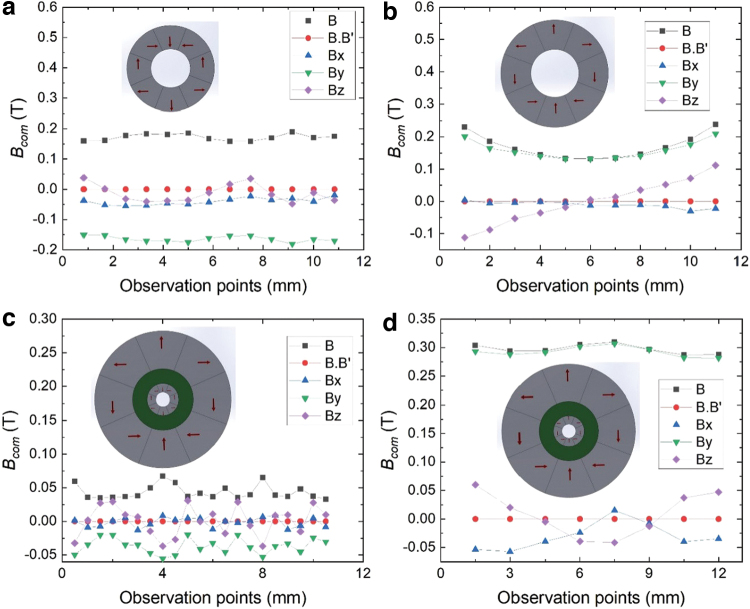
Halbach components and their associated magnetic fields (B) measured at approximate linear distances between 0.25 and 2 mm across the diameters of the Halbach rings: **(a)** inner ring; **(b)** outer ring; **(c)** subtractive mode of a compensated ring; and **(d)** additive mode. See Table 2 for the dimensions of the Halbach arrays. Color images are available online.

Since Equation 3 most accurately estimates the field and size of the Halbach rings, the most convenient method for designing a pair of compensated discs is slicing them out of an already magnetized Halbach ring, a task that is limited by the dimensions of the cutting tool. To address this, two 0.15 T compensated discs were designed with dimensions listed in Table 2. By redesigning the circular disc cutting tool, compensated discs of close to half of the parent solid disc were easily partitioned. The large gap between the discs provided a way to easily mount the compensated disc. The characterization of the individual inner, outer, subtractive, and additive modes of the central field, and its components for the various Halbach rings, are presented in Figure 4. Figure 4a and b shows the magnetic fields and components of the inner and outer Halbach rings, respectively. Figure 4c and d shows, respectively, the minimum and maximum magnetic fields, and components, of the compensated Halbach rings.

**Table 2.** Bore-to-Volume Ratio of Additive Manufacturing Halbach Arrays

Disc	Dimension (mm)	Bore volume (mm^3^)	Magnet volume (mm^3^)	Magnet volume to bore volume ratio
Single disc	$r_{in}=11.5$ $r_{out}=111.5$ Thickness = 25	10,386.90	966,039.75	93
Outer disc/ring	$r_{in}=49.5$ $r_{out}=111.5$ Thickness = 25	192,442.18	783,984.45	4.07
Inner disc/ring	$r_{in}=11.5$ $r_{out}=26.5$ Thickness = 25	10,386.89	41,084.96	3.95

### Electromagnetic simulations of additively manufactured and magnetized Halbach arrays

The magnetic fields of the additively manufactured Halbach cylinders were estimated via electromagnetic (EM) simulations, using magnetic parameters measured from the AM-printed Halbach magnetic materials. To carry out the EM simulations, cylindrical Halbach discs with dimensions corresponding to the fabricated prototypes were designed in solid works Pro 2020 and EM magnetostatic simulations were run using the magnetic properties presented in Figure 4. Specifically, the simulations were executed using an experimentally measured remanence *B_rem_* = 0.45 T, extrinsic coercivity *H_C_* = 11.86 kOe, and permeability = 1.2. The relative permeability of the bonded NdFeB PM was determined using the relation $\mu_{r}=1.06+0.02\left(\frac{BH\left(Max\right)}{MGOe}\right)$^39.^ The simulations for the experiment were conducted using an electromagnetic simulation software (EMS) invoking a magnetostatic study integrated with solid works. The study was performed using a global element size of 1.27 mm per element with a program self-optimized mesh intersection that reduces errors in meshing and allows for an accurate curvature representation that leads to an accurate simulation by adjusting the element size to the curvature of the solid geometry. The materials for the solid geometries were added to the parts and the complete assembly was simulated in air at room temperature with the coercivity directions of each magnet assigned to each part's Cartesian coordinate system in *x* and *y* directions.

**FIG. 5.**
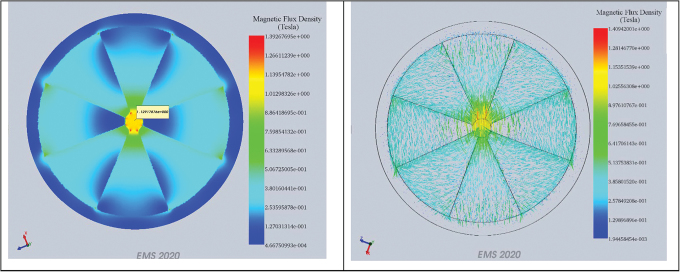
EM simulation results for the parent disc (*left*). Corresponding vector/plot showing the magnetization direction of the different segments (*right*). EM, electromagnetic; EMS, electromagnetic simulation software. Color images are available online.

The central field of both the initial parent solid disc (Fig. 2) and the later fabricated compensated disc pair does not agree closely with experimental results. The measured field for the initial large disc was 0.6 T, whereas the EMS predicted 1.12 T value for the large disc is shown in Figure 5. Similarly, for the large outer disc shown in Table 3, the field was 0.67 T, whereas the measured field was 0.15 T. One source for this discrepancy is structural imperfections, such as voids and misalignment of the Halbach wedges. To confirm this, we scanned one of the initially fabricated large discs on a blue light scanner and observed significant texture in the build layers, as well as noticeable gaps in the section's joints resulting in a misaligned assembly. Based on these observations, we investigated the sizes of Halbach pairs needed to produce the experimentally measured field of 0.15 T and found the dimensions for the simulated set in EMS, as presented in Table 3. The estimated dimensions from the EMS simulation were used to predict fields for the planer geometry using the equation *B* = *B*_rem_ log($\frac{r_{ex}}{r_{in}}$), which agrees well with the simulated fields although the simulation demonstrated about 13% field change with the 50% thickness change of the inner cylinder (not shown). This observation suggests that another reason between the calculated and measured magnetic field values could be the oversimplified estimates of the 3D field in the EM simulation software package. We investigated the reported potential uncertainty from the EM simulation packages and found up to a 28.9% error in the axial component of the diametrically magnetized circular ring.^40^ If we compare the volume difference in the simulated and experimental inner and outer Halbach arrays, ∼75% volume change is observed in the inner array and ∼40% volume difference in the outer array. The outer Halbach array volume change can be attributed mainly to AM imperfections, while the inner rings were attributed to both imperfections and to misalignment of the Halbach sections. Since the Halbach assembly is a more complicated geometry than a diametrically magnetized ring, the discrepancies observed in our simulations are not surprising. In addition, we reproduced the magnetic field and patterns for a Halbach rotor reported in Computers and Solutions (COMSOL) Multiphysics^41^ using EMS simulations. The above results strongly suggest the need of a more sophisticated program that can rigorously account for the geometric volume and demagnetization effects observed in the magnetostatic simulations—although the two-dimensional (2D) field prediction and 2D field distribution patterns of the magnetic field lines are easily handled by the current commercial Multiphysics packages.

**Table 3.** Comparison of the Experimental Dimensions of the Halbach Arrays and EMS-Simulated Sets for the Same Measured Fields

Combination discs	Dimensions	Measured field	EMS-simulated field
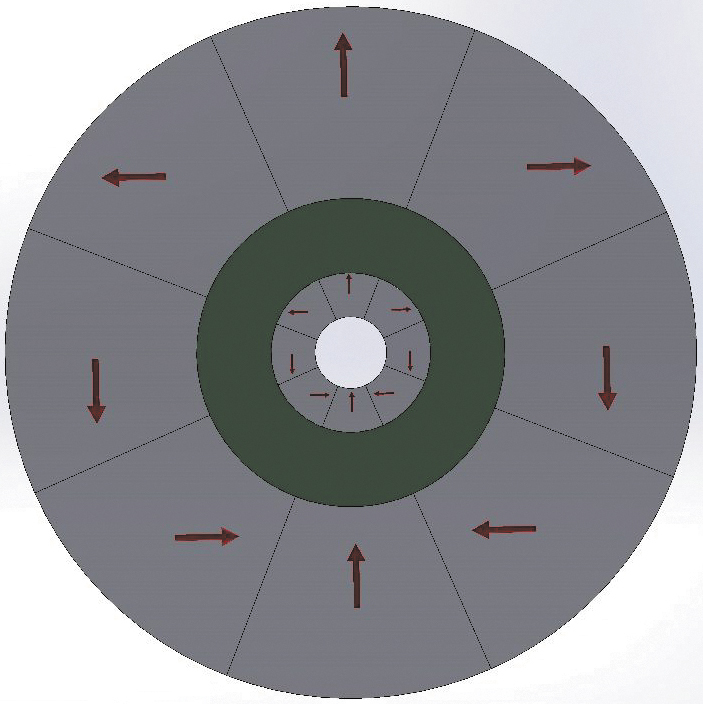 Experimental set	Outer disc ID: 49.5 mm OD: 111 mm	0.15 T	0.67 T
	Inner disc ID: 25.5 mm OD: 11 mm	0.15 T	0.43 T
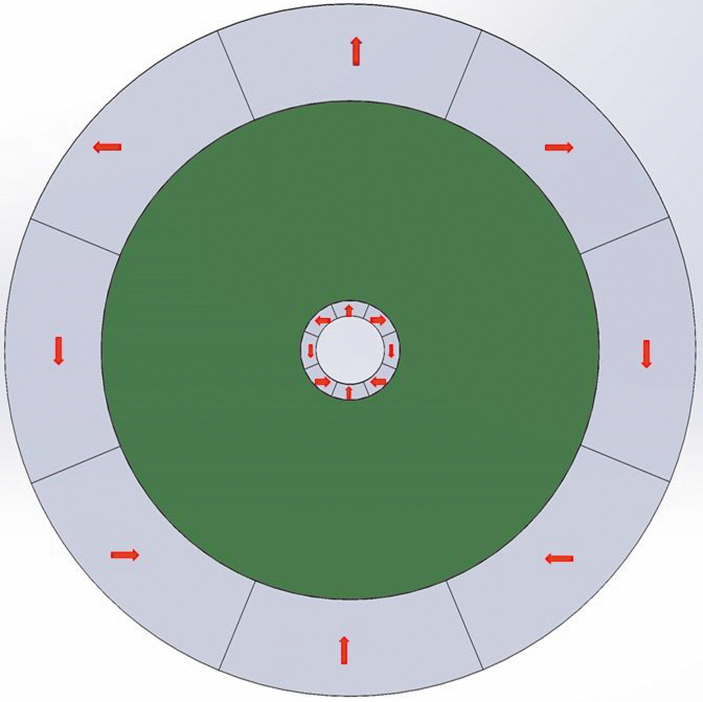 Simulated set in EMS	Outer disc ID: 80 mm OD: 111 mm	N/A	0.15 T
	Outer disc ID: 11 mm OD: 16 mm	N/A	0.15 T

EMS, electromagnetic simulation software; ID, inner diameter; N/A, not applicable; OD, outer diameter.

### Demonstration of conservation of neutron beam polarizability with the compensated Halbach discs

Neutron beams are excellent probes for the study of low atomic-number elements, rich intermetallic compounds, and soft hydrocarbon-rich biological samples, which are otherwise difficult to detect with X-rays and other electromagnetic radiation probes. In the case of specularly reflected neutrons, the scattered intensity pattern of the reflected neutron beam from the thin interfaces is used to study variations of the neutron-optical density, or scattering length density, along the surface normal. However, specular neutron reflectivity can also allow for the extraction of the phase factor, normally lost during the measurement of the scattered signal, enabling the direct inversion of the data into a unique scattering length density or one-dimensional structure.^24^ However, phase recovery requires multiple measurements of the same system, where the index of refraction, or contrast, of some part of the sample can be changed in a systematic way. Traditionally, contrast variation in neutron scattering is achieved by isotopic substitution or the introduction of a magnetic element, in conjunction with the polarized beam.^24–27^ By controlling the vector direction of the magnetic moment in the thin film and the direction of the spin of the polarized neutron relative to the surface normal, we can tune the magnetic contrast between 0 and a maximum given by the internal magnetic induction expressed in Å^−2^.^41^ A nested Halbach array cylinder with the sample of interest located in the center of its aperture has distinct advantages over tradition dipole magnets. Halbach array magnets do not require power supplies or active cooling and the lack of a return field yoke allows for a significantly larger exit scattering angle than traditional resistive dipoles. Moreover, nested ring Halbach design provides both a variable field strength and 2D vector direction control.

**FIG. 6.**
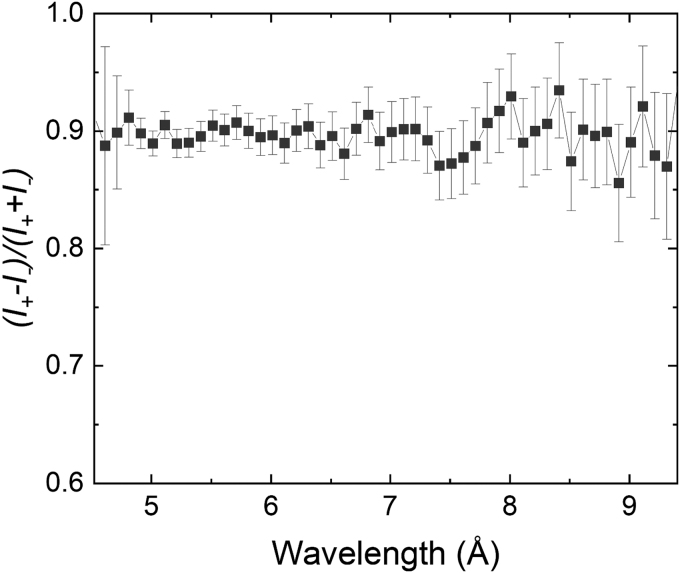
Graph showing the preservation of neutron beam polarization after passing through the Halbach array.

The main concern in using a Halbach array magnet with a polarized neutron beam is the potential that the neutron's polarization will be lost when traversing the magnet. To test this, we placed a Halbach ring magnet at the sample position of the magnetism reflectometer. Before installing the Halbach ring, the incident and exit beam polarizers were determined to have a polarization efficiency greater than 95% using an *m* = 4 polarizing supermirror. The Halbach ring was oriented such that the beam passed parallel to the cylinder axis of the ring, through the center aperture. As shown in Fig. 6, the polarization was then determined by altering the polarization of the incident beam from + to −, while the exit beam polarizer remained in the + state. The polarized beam intensities, *I_+_* and *I_−_* were measured sequentially. The resulting polarization, $\frac{\left(I_{+}-I_{-}\right)}{\left(I_{+}+I_{+}\right)}$, was found to be ∼90% over the measured wavelength range (Fig. 6). The 5% loss in polarization may be retrievable by carefully tuning the guide field on either side of the Halbach ring.

## Conclusions

The first prototype of compensated concentric Halbach array rings was fabricated using FDM-based BAAM technology to generate adjustable magnetic field for the polarized neutron reflectometry. A custom-designed Halbach ring was AM built using PPS-bonded NdFeB PMs and partitioned into a pair of compensated Halbach rings. First tests demonstrated that a 95% polarized neutron beam remains 90% polarized after passing through an AM-fabricated Halbach magnet array. The magnetic field generated by the Halbach array agreed well with the measured and analytically estimated magnetization parameters, and in reasonably good agreement with EM simulations. These measurements show that AM fabrication of well-calibrated Halbach rings can be used effectively to perform magnetic field tunable polarized neutron reflectometry experiments at reflectometers.
